# Novel use of riboflavin as a fluorescent tracer in the dissemination of aerosol and splatter in an open operatory dental clinic

**DOI:** 10.1002/cre2.727

**Published:** 2023-03-31

**Authors:** Morgan A. Emery, Donald Reed, Barbara McCracken

**Affiliations:** ^1^ Department of Clinical Dentistry Southern Illinois University School of Dental Medicine Alton Illinois USA; ^2^ Department of Growth, Development, and Structure Southern Illinois University School of Dental Medicine Alton Illinois USA

**Keywords:** COVID‐19, dental, infection control, riboflavin

## Abstract

**Objectives:**

The rapid spread of severe acute respiratory syndrome coronavirus 2 and the ensuing rise of the COVID‐19 pandemic have impacted healthcare unprecedentedly. With the scarcity of available resources, including healthcare providers themselves, novel methods for tracking aerosol and splatter in real time are required to alleviate demand and increase safety. This study evaluates the utility of riboflavin (vitamin B_2_) as a tracer for splatter/aerosol distribution from ultrasonic scaling in an open operatory clinic.

**Material and Methods:**

In two experimental designs, ultrasonic scaling was performed on 18 volunteers or simulated on a manikin. Riboflavin was introduced into the irrigation system, and aerosol and splatter dissemination were evaluated for both experimental designs.

**Results:**

Ultrasonic scaling utilizing riboflavin solution, in volunteers and manikins, leads to observable particle fluorescence under UV light. Contamination distribution varied across the different suction methods and between the volunteer and manikin trials. Nearly all observed incidences of contamination occurred within the operatory in use.

**Conclusions:**

Riboflavin can be used with minimal risk during dental procedures and allows for the detection of droplet spread in clinical settings in real time.

## 1 INTRODUCTION

In the United States, dental patients and dental healthcare personnel (DHCP) can be exposed to pathogenic microorganisms including *Mycobacterium tuberculosis*, staphylococci, streptococci, human immunodeficiency virus, hepatitis B virus, hepatitis C virus, herpes simplex virus, influenza virus, rhinovirus, and *Legionella pneumonphilia* (CDC, 2003; Ionescu et al., 2020). Transmission can occur through direct contact with patients' fluids (blood, saliva, and gingival crevicular fluid) or indirect contact with contaminated objects (instruments, equipment, or environmental surfaces). More recently, both the Centers for Disease Control and Prevention (2022) and the United States Occupational Safety and Health Administration (OSHA, n.d.) have categorized dentistry as a high‐risk occupation for transmission of severe acute respiratory syndrome coronavirus 2 (SARS‐CoV‐2).

SARS‐CoV‐2 is a coronavirus that causes COVID‐19 and shares significant homology and phylogenesis with other viruses of this family (Z. Chen et al., 2021; Lu et al., 2020). In dentistry, SARS‐CoV‐2 can easily spread from a patient's oral cavity to a new host, through mucosal membranes of the mouth, nose, and eyes, due to prolonged close contact or contact with contaminated objects (Atukorallaya & Ratnayake, 2021). This is mainly due to the presence of SARS‐CoV‐2 in blood and saliva, where presence may be related to the entry of the virus to the oral cavity from the respiratory tract, crevicular fluid, or release of viral particles in the oral cavity via salivary ducts from infected glands (Chmielewski et al., 2021).

While coughing and sneezing can be a problematic means of transmission of microorganisms in a dental setting, the generation of aerosols (particles smaller than 50 µm in diameter) and splatter (particles larger than 50 µm) have become a greater concern (Kumar & Subramanian, 2020). Dental procedures using ultrasonic scalers or high‐speed handpieces are known to generate spatter and aerosol that can be composed of saliva, blood, respiratory fluids, or other organic compounds. The generation of aerosols and splatter becomes particularly challenging as SARS‐CoV‐2 has been reported to maintain viability in aerosol for hours and on surfaces for days (van Doremalen et al., 2020). Thus, maintaining infectious potential and making orally produced aerosol and droplets a high‐risk means of transmission.

Previous studies have frequently focused on utilizing bacterial growth or sodium fluorescein as a tracer. When using bacterial tracers, investigators count aerobic bacterial colonies that grow on agar plates positioned at various locations similar to Ionescu (Gund et al., 2021; Ionescu et al., 2020). However, using a bacterial tracer method does not allow real‐time observation. Instead, the agar plates must be incubated for at least 48 h. Additionally, obligate anaerobic microorganisms, slow‐growing bacteria, and viruses cannot be accounted for using this approach.

In the fields of optometry and otolaryngology, fluorescein has been a primary tracer utilized, but fluorescein has limited use in human subjects due to its potential toxicity. There have been multiple reports of adverse reactions to fluorescein administered topically, intravenously, and orally (Sim et al., 2021). Even with the topical application of fluorescein to detect corneal injury, cases of severe allergic reactions have been reported. Although severe reactions to fluorescein are considered rare, there is currently a lack of literature outlining the upper limit of fluorescein as no LD50 has been established for fluorescein disodium (Dube et al., 2020). Many commercially available fluorescent dyes are not approved for use on humans and many have warnings regarding contact with skin or mucosal surfaces. Additionally, fluorescein sodium can stain clothing, tissue, and plastics if not utilized carefully.

Current published evidence regarding the risk of aerosol and droplet transmission of SARS‐CoV‐2 in dental practices is limited (Allison et al., 2021; Innes et al., 2021). Many of these studies have small sample sizes, limited procedure numbers, or lesser distances from the source of contamination. Additionally, there is also a lack of assessment on extraoral suctioning, usage of safe tracers in human subjects, and reliance on older dissemination studies looking at bacterial settling and growth (Holliday et al., 2021; Nóbrega et al., 2021; Tan et al., 2021; Yang et al., 2021).

Here, we aim to expand existing knowledge of aerosol and splatter dispersion in an open operatory using an in vivo model where we evaluated the feasibility of riboflavin (vitamin B_2_), a water‐soluble vitamin that exhibits fluorescent properties, as a fluorescent aerosol tracer.

## 2 MATERIALS AND METHODS

### 2.1 Study design and data collection for human subjects

This study was conducted with approval from the Southern Illinois University (SIU) Edwardsville Institutional Review Board (protocol 1200). Informed consent was obtained from 18 individuals being provided an ultrasonic dental cleaning at SIU School of Dental Medicine between July 23, 2021 and October 26, 2021. Exclusion criteria were age <18 and >60 years, and self‐reported renal disease, COVID‐19, or other infectious diseases. The sample size was based on six participants per experimental group. Experimental groups included slow suction (≤50 L/min) using a standard saliva ejector (1004092; HIS), high suction (≥100 L/min) using a high‐volume evacuation tip (1126331; ZETES Essentials Healthcare Products), and extraoral suction using an in‐line funnel (6423848; DCI International).

Before the beginning of each procedure, all areas of the study operatory and the operatories immediately adjacent to the study operatory were visually inspected for ultraviolet activity and cleaned with either 70% ethanol or decontaminating wipes to remove any contamination. The written informed consent process and medical history review were then completed, followed by a preoperative exam. Oral opening measurements (vertical and horizontal), plaque and oral hygiene indices, and the total number of teeth were recorded. Participants were divided evenly into three experimental groups, with all receiving standardized dental prophylaxis. The prophylaxis consisted of supragingival scaling using an ultrasonic scaler (Dentsply Sirona Preventative; model #: 8800003) with a lavender tip (82009, Cavitron THINsert Ultrasonic Insert FITGRIP 30 kHz; Dentsply Sirona Preventative) operating at a 30,000 Hz oscillation frequency, removal of residual calculus utilizing hand instruments, full mouth polishing using medium grit prophy paste (220023; Preventative Technologies), and full mouth flossing using waxed teflon floss (84860307; Oral‐B SATINFloss, Procter & Gamble). A dental assistant was utilized during all procedures, providing suction for each experimental group. The solution dispensed from both the ultrasonic scaler and air–water syringe was riboflavin (Millipore Sigma; cat #: R9504) at a 2.5 mM concentration in ultrapure water purified from a Milli‐Q IQ 7003 Water purification system (Millipore Sigma).

Upon completion of prophylaxis, procedure time was recorded and the DHCPs were evaluated for riboflavin contamination using a handheld ultraviolet light with a wavelength of ~440 nm. Examiner and assistant sites of evaluation included arms, chest, mask, and face shields (Supporting Information: Table 1). The operatory was then allowed to remain dormant for 30 min for aerosol settling before inspection. After inspection, the operatory was disinfected.

### 2.2 Experimental setup for manikin

A manikin head equipped with resin teeth (Acadental ModuPRO One M300, Item #: MP_R320) was attached to the dental chair headrest in a standard working position. Ultrasonic scaling occurred using an oscillating frequency of 30,000 Hz with a dental assistant providing the assigned suction type simulating a full mouth cleaning. Upon completion of scaling, evaluations of DHCPs, aerosol settling, and inspection of operatories are observed as described previously.

### 2.3 Operatory

**Figure 1:**
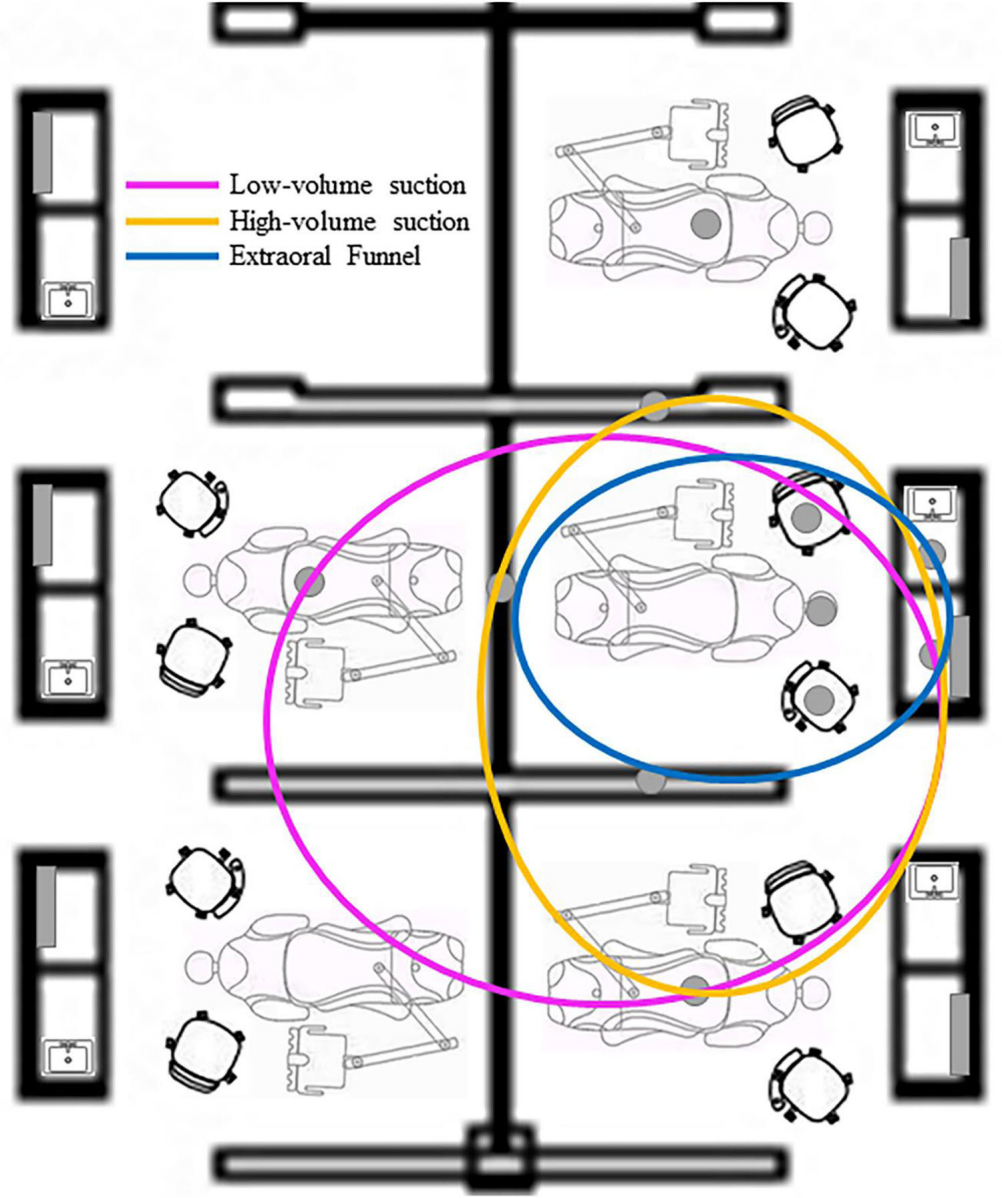
Operatory set‐up diagram showing the further spread of splatter/aerosol measured by suction type.

The researchers used a 2.93 m × 2.62 m × 3.00 m operatory (length, width, and height, respectively) in the main dental clinic of SIU School of Dental Medicine, Alton, IL. The clinic contains open‐air operatories. The study operatory had adjacent operatories on three sides. Equipment included a dental unit (A‐Dec, model #: 511 A), a dental operator stool, a dental assistant stool, and a bench with a sink and computer behind the dental chair. We evaluated both the experimental operatory and adjacent operatories for signs of aerosol or splatter (Figure 1). Both DHCPs wore full personal protective equipment including an N95 mask with overlaying level 3 surgical mask, face shield, gloves, surgical cap, and either a surgical gown or laboratory coat. Participants wore safety glasses and a surgical gown.

### 2.4 Experimental controls

We used multiple internal controls in the experimental design for enhanced result validation. First, the same dentist performed all experimental procedures on human and manikin groups. Data collection was performed by the same group of researchers and assistants for all trials. The same set of operatories was also used for all trials. Finally, a new solution of riboflavin was made before each experiment to minimize degradation and maintain fluorescent potential.

### 2.5 Data analysis

**Figure 2:**
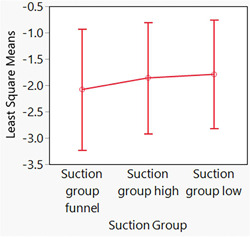
Least squares means plots (and 95% confidence intervals) for the percentages of contaminated patients (the ratio of the number of contaminated patients divided by the total number of patients). Because the logit link function was used for the generalized linear mixed model assessment, these plots use log scales. No difference was demonstrated among groups (*p* = .92).

**Figure 3:**
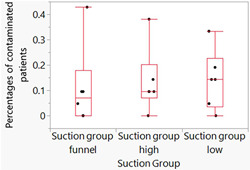
Box plots of raw data for percentages of contaminated patients. To better illustrate the distribution of the data points, they are spread horizontally to minimize their overlapping one another. The ends of the boxes are the 25th and 75th quantiles/quartiles/percentiles. The lines across the middle of the boxes are the medians. The interquartile range is the difference between the quartiles. The lines (whiskers) extend from the boxes to the outermost points that fall within the distance computed as 1.5 (interquartile range).

**Figure 4:**
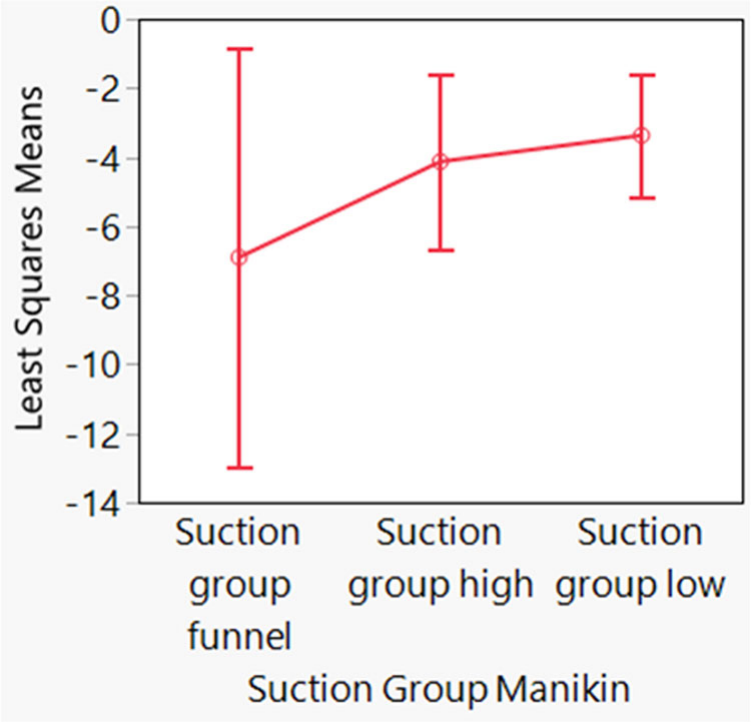
Least squares means plots (and 95% confidence intervals) for the percentages of contaminated manikins (the ratio of the number of contaminated patients divided by the total number of patients). Because the logit link function was used for the generalized linear mixed model assessment, these plots use log scales. No difference was demonstrated among groups (*p* = .42).

Because the assumptions required for traditional linear models are violated if the mean of the response is restricted to a specific range of values, such as a proportion (percentage [%]), statistical assessments were performed using a generalized linear mixed model, with the model effects including fixed effects and random effects (SAS Institute, 2017). Two generalized linear mixed model assessments were performed—one for patients and one for manikins. For patients and manikins, the Suction Group was entered as a fixed effect and had three levels: (1) Suction Group: Low, (2) Suction Group: High, and (3) Suction Group: Funnel. For patients, the patient nested in the Suction Group (patient [Suction Group]) was entered as a random effect, and for manikins, manikin nested in the Suction Group (manikin [Suction Group]) was entered as a random effect. For patients, the outcome (*Y* variable) was a binomial response (the number of contaminated patients and the total number of patients). For manikins, the outcome (*Y* variable) was a binomial response (the number of contaminated manikins and the total number of manikins). For the generalized linear mixed model assessments, a binomial distribution and a logit (log of the odds) transformation were performed [ln(number of contaminated patients/total number of patients) and ln(number of contaminated manikins/total number of manikins)]—that is, a logit link function was used. The residuals resulting from the two mixed model assessments were assessed to determine whether there were serious concerns with the statistical models used and no serious concern was detected. Results are presented in Figures 2, 3–4 and Table 1. The *α* level for assessments was set at .05. Statistical analyses were performed with JMP Pro Statistical Software Release 16.2.0 (SAS Institute, Inc.) and the generalized linear mixed model add‐in released in 2020. Mixed model assessments were based on another recently published book (Hummel et al., 2021) and JMP online webinars (Generalized Linear Mixed Models: Part 4[of 5] and Generalized Linear Mixed Models: Part 5[of 5]).

**Table 1:** Summary statistics table for manikins.

	Suction group manikin																	
	Suction group: Funnel						Suction group: High						Suction group: Low					
	*N*	Minimum	Maximum	Median	25–75 P^a^	Normal distr.	*N*	Minimum	Maximum	Median	25–75 P^a^	Normal distr.	*N*	Minimum	Maximum	Median	25–75 P^a^	Normal distr.
No manikin	3	21	21	21	21.000–21.000		3	20	21	21	20.250–21.000	<0.0001	3	18	21	21	18.750–21.000	<0.0001
Contamination																		
Yes manikin	3	0	0	0	0.000–0.000		3	0	1	0	0.000–0.750	<0.0001	3	0	2	0	0.000–1.500	<0.0001
Contamination																		
Ratio of contamination and total	3	0	0	0	0.000–0.000		3	0	0.0476	0	0.000–0.0357	<0.0001	3	0	0.1	0	0.000–0.0750	<0.0001

^a^ 25–75 P = 25th and 75th percentiles.

## 3 RESULTS

Least squares means plots are presented in Figure 2 for patients and Figure 4 for manikins. Because the logit link function was used for the generalized linear mixed model assessments, these plots are on log scales. Therefore, to help with the interpretation of the data, box plots of the raw data for patients are also presented in Figure 3. Box plots of the raw data for patients are used because the Shapiro–Wilk test indicated that two of the three distributions were nonnormally distributed (*p* ≤ .03). For manikins, the data were nonnormally distributed (*p* < .01); however, median values often overlapped with values the 25th and 75th percentiles; thus, box plots are hard to interpret; therefore, for manikins summary statistics are presented in Table 1. In the captions for Figures 2 and 4, the *p* values for the assessments depicted in the figures are presented.

The results of our analyses indicated little contamination and no difference in contamination (*p* ≥ .42) for the various suction groups (1) Suction Group: Low, (2) Suction Group: High, and (3) Suction Group: Funnel.

The riboflavin tracer exhibited baseline fluorescence in solution, droplets, and as aerosol settled onto hard surfaces during initial evaluation (Figure 1). During 20 min of using the ultrasonic scaler on volunteers, all three types of suction tested reduced noticeable dispersion of fluid particles.

### 3.1 Operatory

The distribution of particles was mostly within a distance of 2 m (Figure 1). Most of the contamination was concentrated around the patient's head for all experimental groups. The furthest distance recorded was 3.45 m in the operatory on the other side of the rear operatory wall. All adjacent operatory walls had at least one incident of contamination. Minimal contamination was also observed on the countertop bench behind the patient's head. The frequency of contamination of operatory areas was low for all experimental groups (Low: *n* = 1, 16%; High: *n* = 2, 33%; Funnel: *n* = 1, 16%).

There were three incidences of contamination in adjacent operatories, with no contamination noted for any trial in the operatory behind the assistant. There was also no noticeable presence of contamination on the operatory light or reaching a height of approximately 1.22 m above the patient.

### 3.2 Clinician and assistant

The outside of the clinician's face shield was the most commonly contaminated site (*n* = 11) with an average distance of 0.457 m. Contamination was observed slightly less frequently on the inside of the clinician's face shield (*n* = 5). The clinician's neck and internal surface of the face shield were the next most common sites (*n* = 5 and 4, respectively). There was no observable contamination on the clinician's arms or chest.

When the clinician and assistant were compared, the clinician had more incidents of contamination (*n* = 22 vs. 14, respectively). This increased number of incidents correlated with a higher contamination rate of the clinician's face shield and neck.

### 3.3 Suction

If suction groups are analyzed in isolation, the inside of the clinician's face shield was contaminated more frequently with low suction (*n* = 4) compared to high (*n* = 0) and funnel (*n* = 2) groups. The inside of the assistant's face shield had a similar contamination frequency (low: *n* = 3; high: *n* = 1; and funnel: *n* = 0). The outside of the clinician's face shield had an increased frequency of contamination for high suction (*n* = 5) when compared to the other groups (low: *n* = 3; and funnel: *n* = 3).

When looking at furthest overall distance of splatter and aerosol spread separated by suction type, the findings suggest there is some difference present (Figure 4). The data suggest that the extraoral funnel may be most effective at reducing splatter and aerosol spread, then the high‐volume suction and the low‐volume suction are the least effective.

## 4 DISCUSSION

Dental aerosol and splatter have been a long‐term concern in dental practices due to their ability to carry pathogens including SARS‐CoV‐2 (Bentley et al., 1994; Harrel & Molinari, 2004; Innes et al., 2021). Contamination from dental procedures has been reported to spread up to 4 m from the source, with aerosols remaining airborne for up to 30 min (Allison et al., 2021; Ionescu et al., 2020; Veena et al., 2015). Droplets and aerosols can be generated from high‐ and low‐speed handpieces, air–water syringes, polishers, and ultrasonic devices (Innes et al., 2021). In the present study, we focused on the dissemination of droplets and aerosol from an ultrasonic scaler, which has been reported to be the main sources of aerosol and splatter generation in a dental practice (Haffner et al., 2021). While other studies commonly used fluorescein, adenosine triphosphate, citric acid, or bacteria as tracers for measuring dispersal, our study is novel in that we are the first to use riboflavin in live patient volunteers (Holliday et al., 2021; Lloro et al., 2021; Puljich et al., 2022; Shahdad et al., 2020; Watanabe et al., 2018).

Riboflavin is a water‐soluble vitamin found in a wide variety of food products with strong absorption in UV and visible spectrums in aqueous solutions (Sheraz et al., 2014). While riboflavin is photosensitive, degradation from visible radiation has been reported to be between 150 and 330 min (Ahmad et al., 2006). It also has an advantage over many other commonly used tracers based on an established safety profile (Sim et al., 2021). Physiologically, riboflavin or vitamin B_2_ is a required coenzyme for multiple cellular biochemical reactions, serving as electron transfer molecules. There is also minimal gastrointestinal absorption and rapid urinary clearance based on its water solubility. In contrast, most fluorescent dyes are not approved for human use and may lead to epithelial tissue damage, anaphylaxis, gastrointestinal issues, or cardiovascular incidents.

The researchers conducted this study in an open operatory clinic, comparing three methods of suction in human volunteers. This allowed the collection of desirable real‐time dissemination data using a relatively harmless tracer in an open operatory clinic. Contamination was primarily found around the patient followed by the areas of the clinician and assistant. Nearly all of the incidents of contamination were found to be within 1.2 m, similar to findings of other ultrasonic studies (Innes et al., 2021; Pierre‐Bez et al., 2021). At the time this study was performed, the dental clinic at SIU School of Dental Medicine was functioning at half capacity due to patient safety concerns during the COVID‐19 pandemic. Patients were being seen by students who were operating in pairs, with one student as the provider and the other student as the assistant, with patients seated only in every other operatory. The findings of this study allowed for the determination to be made that it was safe to return the clinic to full capacity, because the researchers were unable to replicate the volume and distance of splatter and aerosol spread that had been reported in the previously published studies (Allison et al., 2021). This suggests that hygiene techniques utilized on live patients are different from those that were used in the manikin trials and they produce much less splatter and aerosol than was previously reported. Returning the dental clinic to full capacity allowed the dental school to return to providing dental care for the underserved population in Southern Illinois while maintaining patient, student, staff, and faculty safety.

In this study, we observed similar contamination patterns across all suction groups. This is consistent with other reported findings where the use of a saliva ejector (low‐volume suction), high‐volume suction, and extraoral suction results in a reduction of ≤96% of particles generated (Lloro et al., 2021; Ou et al., 2021; Puljich et al., 2022). When comparing saliva ejector to high‐volume suction contamination, the difference was found to be minimal between these groups (D'Antonio et al., 2022; Holloman et al., 2015; Melzow et al., 2022; Yang et al., 2021). These results were in contrast to the recommendations that reducing ultrasonic speed correlates with contamination of the operatory (Shahdad et al., 2020). Additionally, our data provides some evidence that the extraoral funnel suction technique may be the most effective at reducing splatter and aerosol spread, but it may be beneficial to repeat the project with a larger sample size to confirm this finding (Figure 1).

Furthermore, we also suggest similarly to Shahdad et al. (2020), that clinical experience and dental instrument positioning is critical for reduced aerosol exposure and generation. During our study, our clinician used a 2 × 2 piece of cotton nu gauze placed on the inner edge of the lower lip or upper lip when using the ultrasonic scaler or hand instruments on the facial or lingual of the lower anterior teeth and upper anterior teeth, respectively. The piece of gauze collects any water spray directed out of the mouth and minimizes water spray on to the external portion of the patient's face. Additionally, during polishing, our clinician wiped off the prophy cup head of any saliva and residual prophy paste before reloading with fresh prophy paste. This practice minimizes splatter coming off of the prophy angle during polishing. The clinician established both of these techniques during their years in clinical practice and is further supported by I.‐H. Chen et al. (2022) who found proficiency and experience lead to reduced splatter distance. Moreover, suction was maintained at all times while ultrasonic scaling was being performed, with the assistant suctioning at exact intraoral locations for high volume and saliva ejector, or maintaining a funnel at approximately 2.5 cm away from the volunteer's mouth.

Finally, the ability to safely utilize riboflavin as a fluorescent tracer in live patients has potential uses in dental and medical practice and education. Introducing riboflavin to the water in the dental unit provides a method to qualitatively measure a student's improved performance in their infection control practices over time and a way to attach a grade to that qualitative improvement. Additionally, riboflavin could be used as a safety mechanism to address potential pathogen spread and suction efficiency in Intensive Care Units and other hospital or medical practice settings.

### 4.1 Limitations

Our study has several limitations, and our results must be interpreted with these in mind. Although it was easy to distinguish the presence or absence of particles through UV light inspection, we did not analyze fluorescent intensity or riboflavin concentrations. Additionally, under UV inspection care had to be taken to avoid false positives due to the fluorescence of prophy paste on the operatories. Thus, analysis of splatter was more tangible than aerosols. Finally, our study included only a single dental procedure, excluding multiple other dental procedures that are known to generate contamination. However, this may be less of a limitation for determining riboflavin's feasibility as a tracer based on a report that most microbiota in particles comes from dental irrigant rather than saliva (Meethil et al., 2021). Additionally, clinic personnel will have reduced exposure risk due to salivary pathogen dilution during dental procedures (Allison et al., 2021).

Future studies might include repeating the study design with a larger sample size, without an assistant present, and with a less experienced provider or multiple different providers. Another recommended future study would be to repeat the study design utilizing cellulose filter paper to allow for assessment of fluorescent intensity or riboflavin concentrations to provide more qualitative results when comparing differences between suction techniques.

## 5 CONCLUSION

Within the limitations of this exploratory study, our data suggest the following best practices:

1. Use of an extraoral funnel suction appears to maximize reduction in aerosol and splatter spread but additional research with a larger sample size needs to be conducted to confirm these findings.
2. Utilizing a live assistant to add intentional suctioning on all aerosol‐producing procedures to maximize splatter and aerosol reduction, especially in scenarios where there is a concern about spread of an infectious agent, such as in future pandemics.
3. Use of live patients to confirm dental best practices is recommended as device or equipment settings that can be used on manikins may not have direct real‐world applications.

Finally, we found that riboflavin can be used to detect droplet splatter using UV light. As riboflavin is widely available and relatively harmless to patients, it can be used during routine procedures to help clinical personnel with training and decontamination for the reduced spread of infectious diseases.

## AUTHOR CONTRIBUTIONS

Morgan A. Emery contributed to data acquisition, analysis, interpretation, drafted, and critically revised the manuscript. Donald Reed and Barbara McCracken contributed to the conception, design, data acquisition, analysis, and interpretation, drafted, and critically revised the manuscript. All authors gave final approval and agree to be accountable for all aspects of the work.

## CONFLICT OF INTEREST STATEMENT

The authors declare no conflict of interest.

## Supporting information

Supporting information.

## Data Availability

Data are available in article in the Supporting Information material.
